# Disease Monitoring and Health Campaign Evaluation Using Google Search Activities for HIV and AIDS, Stroke, Colorectal Cancer, and Marijuana Use in Canada: A Retrospective Observational Study

**DOI:** 10.2196/publichealth.6504

**Published:** 2016-10-12

**Authors:** Rebecca Ling, Joon Lee

**Affiliations:** ^1^ School of Public Health and Health Systems Faculty of Applied Health Sciences University of Waterloo Waterloo, ON Canada

**Keywords:** public health informatics, Internet, information seeking behavior

## Abstract

**Background:**

Infodemiology can offer practical and feasible health research applications through the practice of studying information available on the Web. Google Trends provides publicly accessible information regarding search behaviors in a population, which may be studied and used for health campaign evaluation and disease monitoring. Additional studies examining the use and effectiveness of Google Trends for these purposes remain warranted.

**Objective:**

The objective of our study was to explore the use of infodemiology in the context of health campaign evaluation and chronic disease monitoring. It was hypothesized that following a launch of a campaign, there would be an increase in information seeking behavior on the Web. Second, increasing and decreasing disease patterns in a population would be associated with search activity patterns. This study examined 4 different diseases: human immunodeficiency virus (HIV) infection, stroke, colorectal cancer, and marijuana use.

**Methods:**

Using Google Trends, relative search volume data were collected throughout the period of February 2004 to January 2015. Campaign information and disease statistics were obtained from governmental publications. Search activity trends were graphed and assessed with disease trends and the campaign interval. Pearson product correlation statistics and joinpoint methodology analyses were used to determine significance.

**Results:**

Disease patterns and online activity across all 4 diseases were significantly correlated: HIV infection (*r*=.36, *P*<.001), stroke (*r*=.40, *P*<.001), colorectal cancer (*r*= −.41, *P*<.001), and substance use (*r*=.64, *P*<.001). Visual inspection and the joinpoint analysis showed significant correlations for the campaigns on colorectal cancer and marijuana use in stimulating search activity. No significant correlations were observed for the campaigns on stroke and HIV regarding search activity.

**Conclusions:**

The use of infoveillance shows promise as an alternative and inexpensive solution to disease surveillance and health campaign evaluation. Further research is needed to understand Google Trends as a valid and reliable tool for health research.

## Introduction

With an increasing number of people using the World Wide Web, their activities generate “big data” and provide meaningful research in infodemiology, which is the study of patterns and determinants of information on the Web or in a population with the purpose to inform public health and public policy [1]. Infodemiology is of two types, supply based and demand based, which are contrasted by their focus on the provision or uptake of information, respectively. Supply-based infodemiology examines the amount and quality of information available on the Web, such as the number of websites, media reports, and blogs related to vaccinations as well as the level of quality (eg, positive or negative portrayals of vaccines) [2]. On the contrary, demand-based infodemiology examines the patterns of people’s search behaviors for seeking information on the Web, such as the increase in search activities in light of related news reports [2]. Furthermore, a specific branch of demand-based infodemiology known as infoveillance is the observation of information seeking behaviors on the Web. Notably, the use of Web-based data heavily relies on crowdsourcing from the public and their engagement online. Similarly, other applications such as digital disease detection and surveillance work on a similar basis to effectively utilize the continuous generation of Web-based data from this growing digital age [3,4]. However, in this study, the applications of infoveillance in health research are specifically evaluated.

Infoveillance has proven to be successful in predicting infectious disease outbreaks, spawning the development of Google Flu Trends [2,3]. Google Flu Trends leverages the principle that changes in information and communication patterns portray early “symptoms” of a disease emerging in a population [1]. There is strong evidence regarding the predictive utility of search activity and infectious disease outbreak from digital epidemiologic studies. Milinovich et al [4] identified 17 infectious diseases that were positively correlated with search activity trends on Google, with a majority being vaccine-preventable, vector-borne, and sexually transmitted diseases. Most recently, the Colombian Zika virus outbreak showed positive association with search activities made on Google [5]. Current research in infoveillance has primarily focused on infectious diseases, but less is known about its utility pertaining to chronic diseases [6]. In a systematic review looking at the applications of Google Trends in health research, among the 70 included studies, most studies focused on general population surveillance (33%), followed by infectious diseases (27%), mental health and addictions (24%), and noncommunicable diseases (16%) [6].

With the growing digital era, many people now turn to the Web to learn about disease symptoms, diagnosis, and treatments, such as various cancers [7]. One study surveyed more than 12,000 individuals from 12 countries and reported that more than 45% of respondents sought health information on the Web to self-diagnose their condition [8]. Furthermore, these Web-based information seeking behaviors may be more common among diseases that are highly stigmatized in a society, owing to the perception of the disease, to deter from seeking professional help [9]. Thus, such Web-based search activities can provide valuable insights about the prevalence and distribution of chronic diseases. Disease search trends have been studied for breast cancer [10,11], lung cancer [12], status epilepticus [7], transient ischemic attack [13,14], and kidney stones [15]. Regarding stigmatized diseases, research is available on dementia [16], mental health [9], and suicide deaths [17,18]. Overall, these studies reported moderate to strong degree of associations, suggesting a potential value in applying infoveillance to chronic diseases. Foroughi et al [19] found that cancer-related terms were associated with Web search activity for the burden of cancers, such as cancer incidence and mortality, in Australia, New Zealand, the United Kingdom, Canada, and the United States. Also, an American study showed associations between suicide rates and Google search trends relating to “how to suicide,” “commit suicide,” and “suicide prevention” among all 50 states in 2009 [18]. By applying key search terms associated with the disease outcome, search activity trends may be used to portray further insights about the population health and behaviors. However, in comparison with the numerous studies done on infectious diseases, further research is needed to validate the use of infoveillance for chronic diseases.

Second, infoveillance has also been used to monitor and track the success of marketing campaigns measured by the generation of interest and activity of a population observed on the Internet [2]. Traditional epidemiologic tools, such as nationwide surveys, cohort studies, or registries, are often labor and resource intensive. Because of these factors, it could be very difficult and impractical to observe population trends happening before, during, and after the campaign period. Thus, infoveillance offers an advantage and practicality over traditional methods in campaign evaluation. Currently, few studies have investigated public health campaigns and their effects on Web searches. The most commonly studied campaign is the Breast Cancer Awareness Month campaign established in the United States. These studies reported that the Breast Cancer Awareness Month campaign was effective in triggering search activity [10,11,20]. Swenson and Lindblom [21] analyzed Google Trends for “breast cancer,” “colon cancer,” and “cervical cancer” and reported that cancer awareness month campaigns, specifically breast cancer and colon cancer, increased public awareness as shown by increased Web search activities. In the Republic of Ireland, Davis et al [22] found a direct association between media campaigns for erectile dysfunction and related search activity patterns. Despite limited research on the influence of health campaigns on search activity, other studies showed that media events including news reports and announcements regarding public figures also prompted health seeking behaviors [22-24]. These findings suggest potential value for assessing search activity trends; however, further research is needed in this area to evaluate the effectiveness of infoveillance for monitoring health campaigns.

Thus, the aim of this observational study was to explore the applications of infoveillance in information seeking behavior for human immunodeficiency virus (HIV) and acquired immunodeficiency syndrome (AIDS), stroke, colorectal cancer, and marijuana use. Using Google Trends, these search activities were assessed against census data and public health campaigns to examine their relationships. To the best of our knowledge, these diseases have not been studied, particularly in the Canadian context. We first hypothesized that search trends are associated with the disease patterns of a population. Second, we hypothesized that the launch of the health campaign stimulates Google search activity, shown on Google Trends. The outcomes of this research provide new insights for public health professionals and contribute to further understanding of infodemiology in health research.

## Methods

### Study Design

In this retrospective study, search activity on Google was examined for colorectal cancer, HIV and AIDS, stroke, and marijuana use from 2004 to 2015 in Canada. Google Trends provided relative search volumes (RSVs) for particular search queries. First, to study the effectiveness of infoveillance in chronic disease monitoring, the search activities were compared against disease prevalence on an annual basis between 2004 and 2015. Second, to investigate the application of infoveillance in health campaign evaluation, the levels of search activity before, during, and after the campaign were analyzed.

### Selection of Health Campaigns

Health campaigns were sought through peer-reviewed sources and gray literature. The search was narrowed to identify campaigns in Canada and campaigns implemented after 2004 because Google Trends provides its data only after this time point. Health campaigns through any medium, such as televised advertisements, program delivery, or pamphlet distribution, were considered in the assessment. However, the minimum data elements required for this study were the campaign duration, frequency, and location. Health campaigns that met these criteria were then screened based on their disease focus. Preference was given to diseases that had not yet been reviewed in current literature, as well as chronic diseases in order to examine the infodemiology applications for chronic disease monitoring. Thus, health campaigns were chosen based on their transparency and availability of public information, which subsequently dictated the 4 diseases studied in this paper.

As a result, this includes the “ColonCancerCheck” campaign led by the Government of Ontario, “End HIV Stigma” campaign led by the Positive Living Society of British Columbia (formerly known as the British Columbia Persons With AIDS Society), “Anti-Marijuana” campaign led by the Government of Canada, and “Make Health Last” campaign led by the Heart and Stroke Foundation. Because these campaigns differ in purpose, duration, and delivery channels, this may help identify components of a successful campaign that lead to increased information seeking behaviors. Table 1 provides a summary and comparison of the campaign features.

### Data Collection

#### Search Activity Given by Google Trends

In 2012, Google Search accounted for 78% of the global market share among all search engines [30]. Aptly, its huge popularity will provide the best portrayal of online search activity in the Canadian population. Google Trends is a publicly available, free analytical tool that provides aggregated search results since 2004. The disease name was used as the base of each search term (ie, “colorectal cancer,” “HIV/AIDS,” “marijuana use,” and “stroke”). Furthermore, to produce an accurate portrayal of the search activity behaviors of the population, the related key terms suggested by Google Trends (see Multimedia Appendix 1) were reviewed for inclusion in the search. These suggested terms helped to capture the most popular search queries as well as synonymous terms. However, terms that were ambiguous, carried multiple meanings, and/or related to disease statistics were excluded from the data collection. For example, recommended Google searches such as “Hiv/aids in Africa,” “2 stroke,” “heart and stroke lottery,” and “hiv prevention” did possess the target word in the query but contextually were not relevant to the study and were subsequently not collected in this process. Using Boolean operators, the search query contained the keywords relevant to the disease in order for Google Trends to generate the aggregate search patterns. The final search queries used in this study are provided in Table 2. Google Trends returned results in RSV, which facilitates comparisons between terms. The RSV is defined as the quotient of the number of searches at a specific time over the total number of searches in a period of time, for a specific geographic location. Essentially, the RSV represents the popularity of the search term at a specific point in time. After narrowing filters in the Google Trends tool, weekly RSV data were exported for the period February 2004 to December 2015 in Canada and the specific Canadian provinces that were associated with the campaigns.

**Table 1:** Summary of health campaigns.

Campaign features	Disease interests			
	Colon cancer	HIV^a^ and AIDS^b^	Marijuana use	Stroke
Organization	Government of Ontario	Positive Living Society of British Columbia	Government of Canada	Heart and Stroke Foundation
Campaign name	“ColonCancerCheck”	“End HIV Stigma”	“Anti-Marijuana”	“Make Health Last”
Purpose	Increase colon cancer screening practices [25]	Reduce stigma surrounding HIV [26]	Educate about the negative health consequences of marijuana among adolescents [27]	Increase awareness of stroke [28] Educate about risk assessment [28] Promote health [28] Encourage donations [28]
Delivery channels	Health care provider referrals as well as television advertisements, radio announcements, newspaper advertisements, and pamphlets across Ontario [25]	30-second public service announcements shown on 40 participating radio and television stations in British Columbia [26]	Television, Web-based advertisements, and social media [27]	Canadian Broadcasting Corporation (CBC) platforms including CBC television, CBC networks, CBC Player, regional stations, and digital banner [29]
Campaign period	April 2008 to September 2008 [25]	July 2006 to July 2007 [26]	October 2014 to December 2014 [27]	February 2013 to May 2013 [29]
Duration	6 months	12 months	3 months	4 months

^a^HIV: human immunodeficiency virus.

^b^AIDS: acquired immunodeficiency syndrome.

**Table 2:** List of search terms.

Search query filters	Colorectal cancer	HIV^a^/AIDS^b^	Marijuana use	Stroke
Search terms	Colorectal cancer + colorectal diagnosis + colorectal screening + colorectal cancer screening + colon cancer + colon cancer symptoms	Hiv + aids + human immunodeficiency virus + acquired immunodeficiency virus + hiv symptoms + hiv diagnosis + aids symptoms + aids diagnosis + hiv contraction	Marijuana use + drug abuse + marijuana side effects + marijuana effects + effects of marijuana + drug use+ drug addiction	Stroke + stroke symptoms + stroke onset
Geographic locations studied	Canada and Ontario	Canada and British Columbia	Canada and Ontario	Canada and Ontario
Period of data collection	February 2004 to December 2015	February 2004 to December 2015	February 2004 to December 2015	February 2004 to December 2015

^a^HIV: human immunodeficiency virus.

^b^AIDS: acquired immunodeficiency syndrome.

#### Disease Statistics

Statistics on the diseases were searched in both open peer-reviewed journals and gray literature sources. Data reported on the disease prevalence were preferred over disease incidence; however, if prevalence data were not available, then disease incidence data were still used. The disease incidence and prevalence data were obtained from Statistics Canada and other governmental publications for the period 2004 to 2014.

### Data Analysis

#### Relationship Between Search Activity and Disease Patterns

Data trends for each disease were graphed together to compare disease search activity and disease prevalence or incidence. Disease monitoring was first assessed via visual inspection to identify patterns in the data. A subsequent Pearson correlation analysis was conducted in IBM SPSS Statistics 23(IBM Corporation) to detect statistical significance. The mean annual search activity was plotted against the annual disease rates to determine any correlations. This procedure was repeated for all 4 diseases.

#### Impact of Health Campaign on Search Activity

To evaluate the public health campaign effect on search activity, the joinpoint methodology was used [11,31]. This analysis was used by Schootman et al [11] to investigate the impact of cancer diagnosis and deaths of public figures on search behaviors on Google. Similarly, in the time series of Google search activity, the joinpoint methodology tested whether weekly changes in RSV around the time of the campaign were statistically significant. This was completed by fitting linear regression lines to the natural logarithm of the weekly RSV. Inflection points were identified to examine any significant differences between the slopes of the linear regression lines. Analyses were performed to examine online activities 20 weeks before the campaign, during the campaign, and 20 weeks after the campaign. Secondary analyses were conducted to examine shorter segments of 5, 10, and 15 weeks before, during, and after the campaign. The purpose of assessing different periods was to identify any potential effects of the health campaign on increasing people’s search behavior on the Web. For example, examining the 5 weeks leading up to the campaign and the 5 weeks following the campaign detected any immediate change caused by the campaign implementation. However, because RSV data were collected on weekly measures, the number of observations tested by the joinpoint program was low, affecting the statistical power. Therefore, longer-term trends were investigated in 10-, 15-, and 20-week segments. A maximum of 3 joinpoints were fitted to each RSV time series. This maximum was chosen because of the predicted pattern we expected to see. The first joinpoint would be observed at the start of the campaign period because we hypothesized an increase in search activity. Following that surge, the search activities would peak during the campaign period and slightly decline over time, generating the second joinpoint. The third joinpoint would be observed at the end of the campaign period, in which search activity would return to baseline.

## Results

### Internet Search Activity and Disease Prevalence

**Figure 1:**
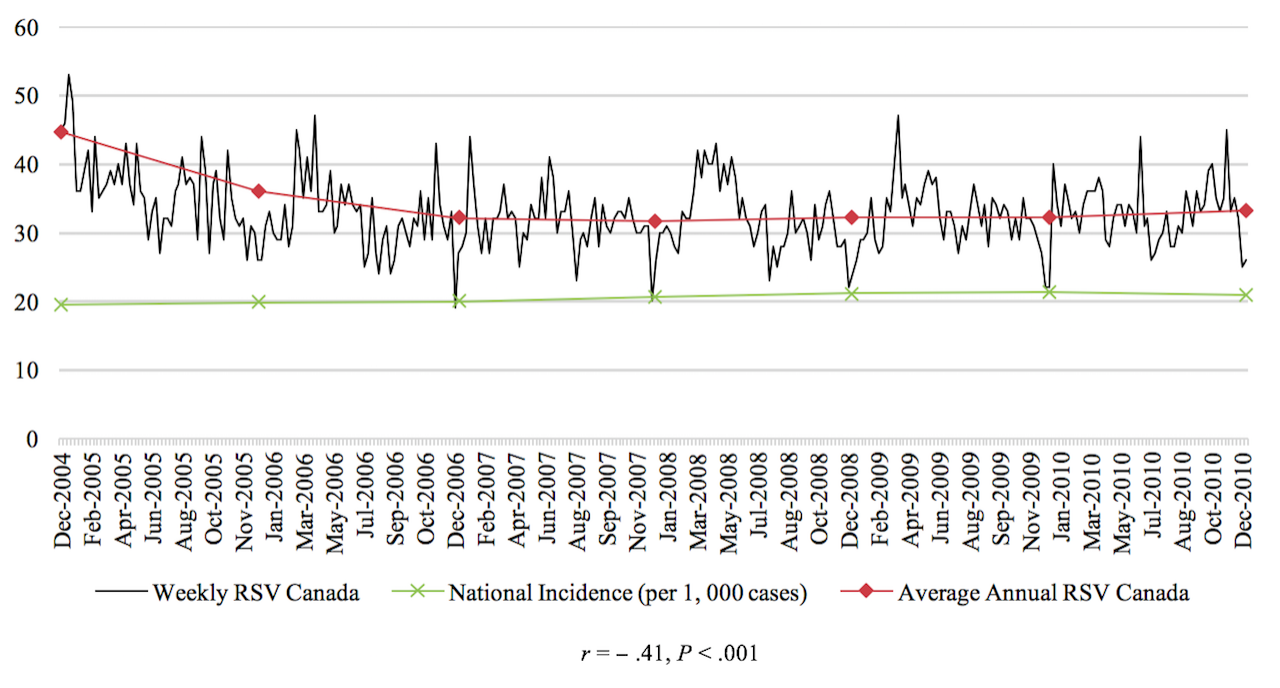
Web-based search activity and incidence trends for colorectal cancer. RSV: relative search volume.

**Figure 2:**
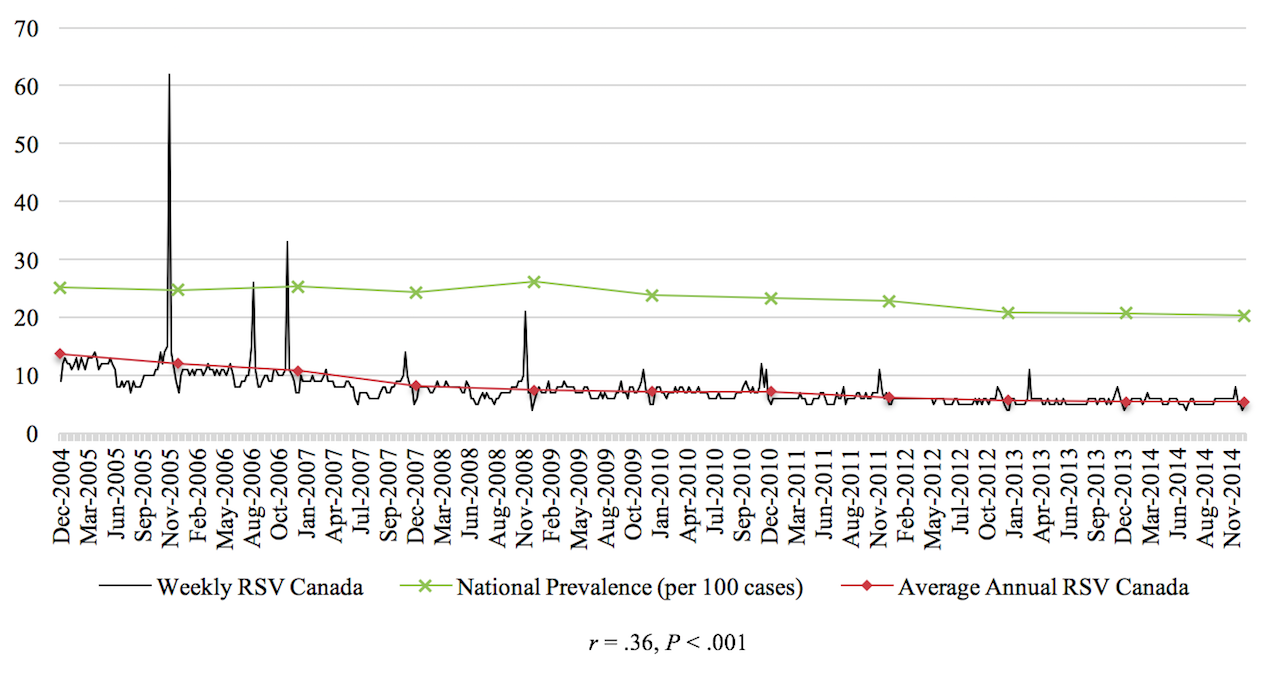
Web-based search activity and prevalence trends for human immunodeficiency virus. RSV: relative search volume.

**Figure 3:**
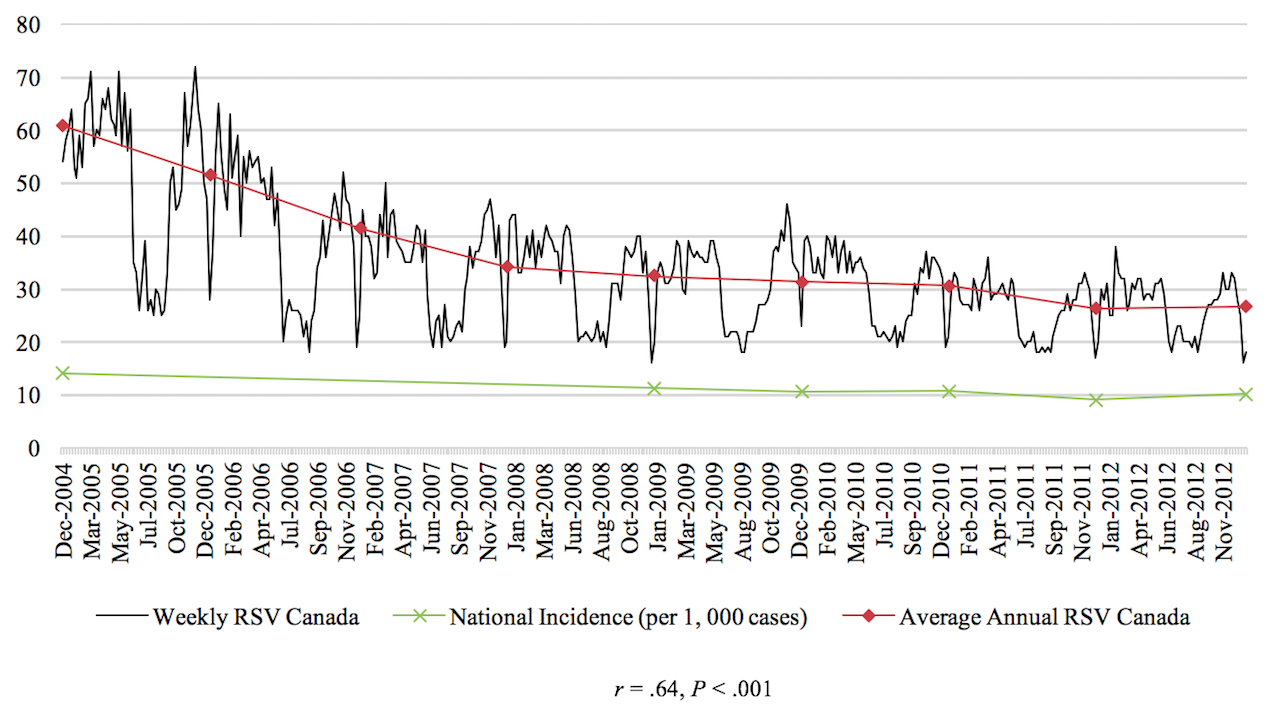
Web-based search activity and incidence trends for marijuana use. RSV: relative search volume.

**Figure 4:**
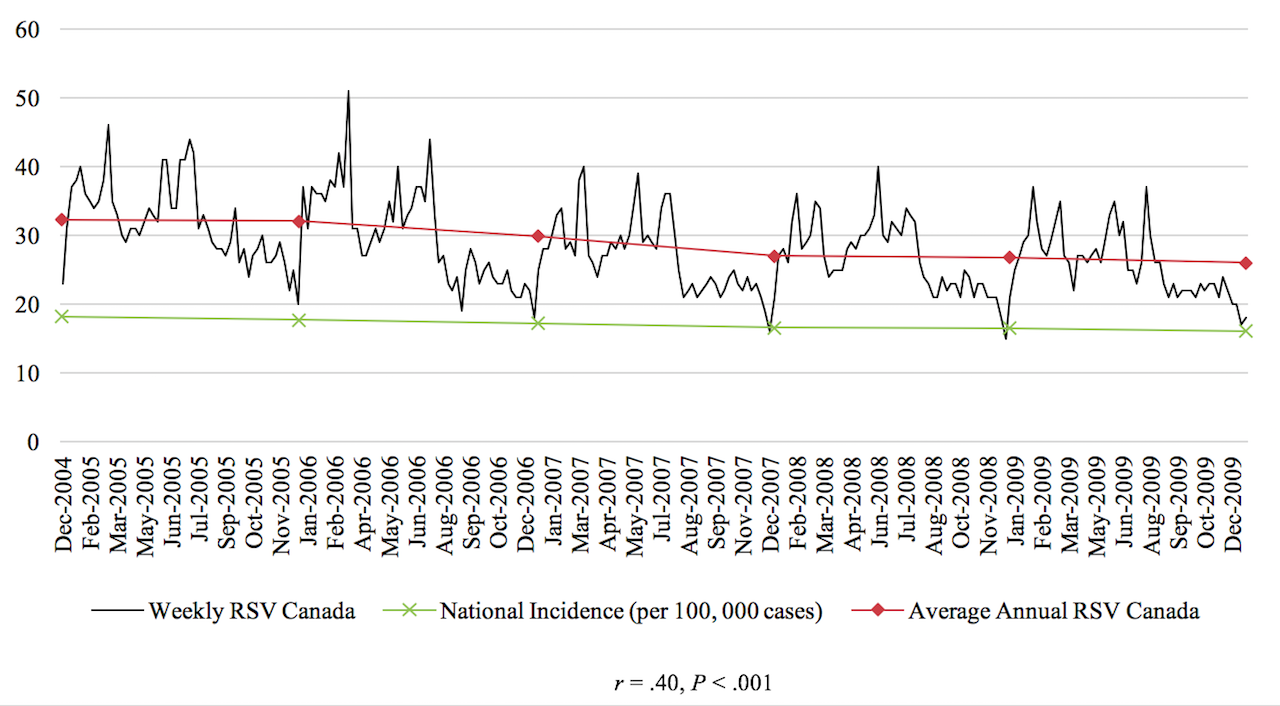
Web-based search activity and incidence trends for stroke. RSV: relative search volume.

The Pearson correlations tested the relationship between RSV of the disease and the prevalence or incidence of the disease in a given time period for the 4 diseases studied in this paper. In general, the relationship between these 2 variables was significant for each of the 4 diseases. From 2004 to 2010, there was a negative correlation between colorectal cancer incidence and search activity (*r*=−.41, *P*<.001). The association between HIV search activity and incidence data was moderately positive between 2004 and 2014 (*r*=.36, *P*<.001). The prevalence of marijuana use pertaining to 2004 and from 2008 to 2012 was positively correlated with search activity (*r*=.64, *P*<.001). Finally, from 2004 to 2009, the association between the search activity of stroke and the incidence was moderately positively correlated (*r*=.40, *P*<.001). Figures 1-4 depict the Web-based search activity trend in weekly RSV values and the calculated average RSV values for the year compared with the disease rates for colorectal cancer, HIV infection, marijuana use, and stroke.

### Overview of Health Campaigns

#### Visual Assessment of Campaign Impact on Internet Search Activity

**Figure 5:**
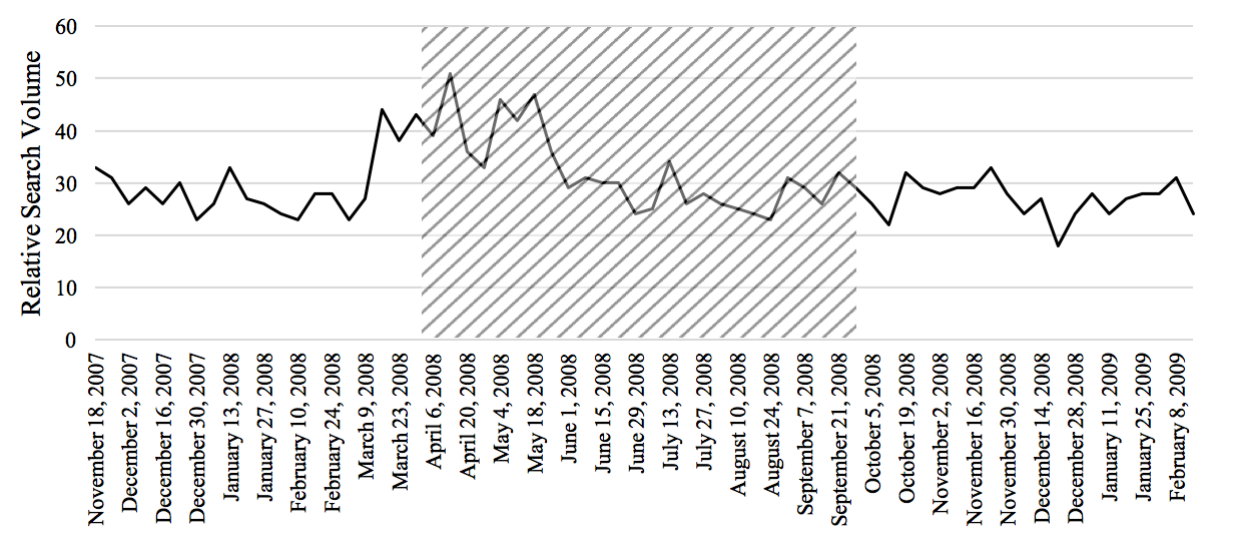
Weekly Web-based search activity for colorectal cancer before, during, and after the campaign period. Highlighted section depicts the campaign duration.

**Figure 6:**
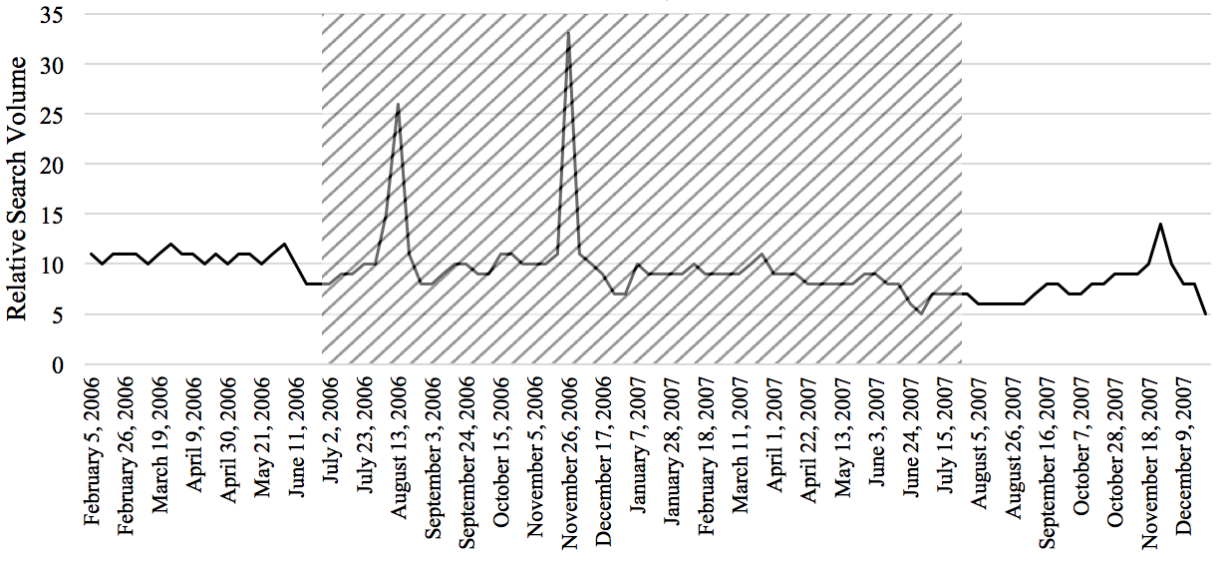
Weekly Web-based search activity for human immunodeficiency virus before, during, and after the campaign period. Highlighted section depicts the campaign duration.

**Figure 7:**
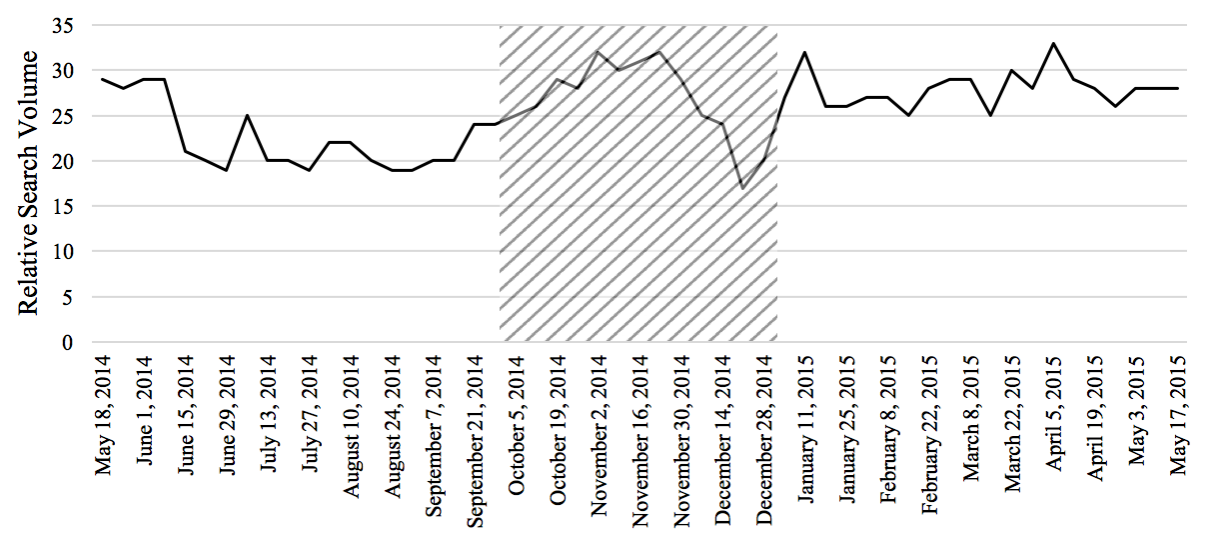
Weekly Web-based search activity for marijuana use before, during, and after the campaign period. Highlighted section depicts the campaign duration.

**Figure 8:**
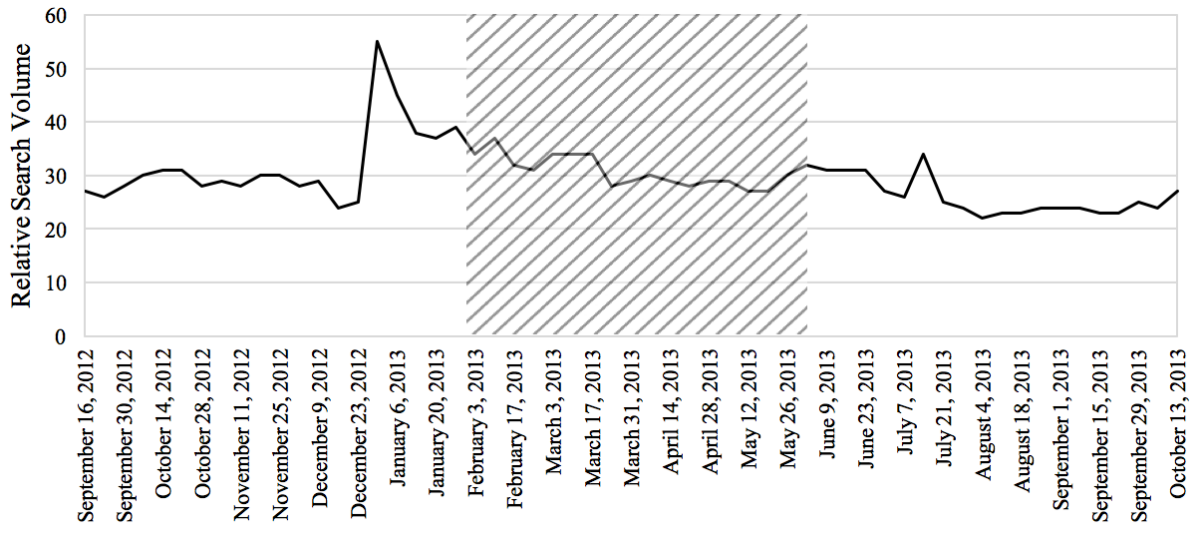
Weekly Web-based search activity for stroke before, during, and after the campaign period. Highlighted section depicts the campaign duration.

The RSVs of the 20 weeks before the campaign, during the campaign period, and 20 weeks after the campaign were graphed to visually inspect them for increases and decreases in search activity caused by the implementation of the campaign (examples shown in Figures 5-8). On the basis of this preliminary assessment, the “Anti-Marijuana” campaign (Figure 7) shows the largest increase in search activity during the campaign period than the other 3 campaigns. This is shown by the increase in activity as the campaign is introduced and the decrease in activity near the end of the campaign. No definitive inference could be made about the impact on information seeking behavior for the “Make Health Last” campaign for stroke and “End HIV Stigma” campaign.

#### Statistical Findings of Campaign Impact on Internet Search Activity

**Figure 9:**
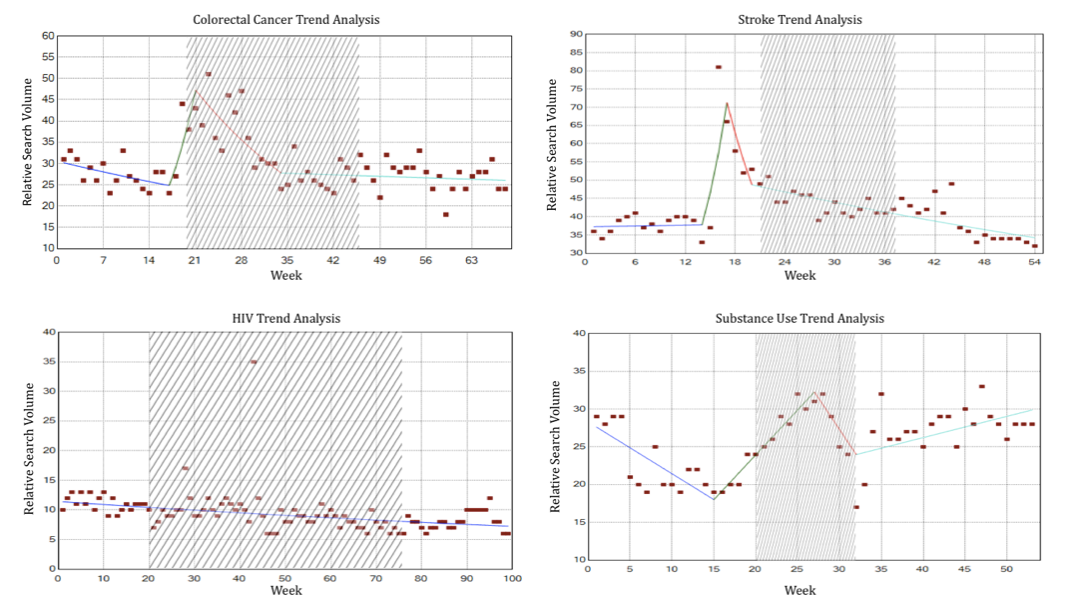
Joinpoint analysis of the 4 diseases studied. The highlighted area shows the period the campaign was in effect. HIV: human immunodeficiency virus.

Figure 9 shows the results from the joinpoint analysis. The results for “ColonCancerCheck” suggest strong effect of campaign implementation on Google search activity. A significant inflection point, which means a statistically significant change in slope, was detected at week 21. This point is very close to the 20-week mark of when the campaign began in our analysis. Notably, Figure 9 shows a declining trend of search activity between weeks 1 and 17 before experiencing a steep increase in search activity around the same time point of campaign implementation. The “Anti-Marijuana” campaign was the only campaign that showed an increase in search activity during the period of the campaign. The joinpoint analysis calculated a significant inflection point at week 16 in which search activity changes from a downward trend to an upward trend. The implementation of the campaign may have contributed to this observed increasing Google search activity for marijuana information. No significant associations between the campaigns and online search activity were seen for the “Make Health Last” and “End HIV Stigma” campaigns. The inflection points were not closely associated with the implementation of the campaign. Table 3 provides a summary of the statistical findings. A 20-week segment is provided as main results because it contains the most data points in its analyses; however, Multimedia Appendices 2-5 provide the statistical findings for the 5-, 10-, and 15-week period assessments.

**Table 3:** Joinpoint analysis for the periods 20 weeks before, during, and 20 weeks after the campaign.

Statistical outputs		Colorectal cancer	HIV^a^	Marijuana use	Stroke
**Segment 1 (week)**		1-17	1-99	1-16	1-14
	Slope, RSV^b^/week (95% CI)	−1.21 (−2.7 to 0.3)	−0.46 (−0.6 to −0.3)	−2.72 (−4.1 to −1.3)	0.10 (−1.0 to 1.2)
	*P* value^c^	.11	<.001	<.001	.85
**Segment 2 (week)**		17-21	—	16-28	14-17
	Slope, RSV/week (95% CI)	17.46 (−2.8 to 41.9)	—	4.93 (2.7 to 7.3)	23.55 (−1.0 to 54.3)
	*P* value^c^	.09	—	<.001	.06
**Segment 3 (week)**		21-34	—	28-33	17-20
	Slope, RSV/week (95% CI)	−4.01 (−6.2 to −1.8)	—	−6.52 (−14.4 to 2.1)	−11.89 (−29.4 to 10.0)
	*P* value^c^	<.001	—	.13	.26
**Segment 4 (week)**		34-68	—	33-55	20-54
	Slope, RSV/week (95% CI)	−0.19 (−0.7 to 0.3)	—	1.19 (0.4 to 2.0)	−1.03 (−1.3 to 0.8)	
	*P* value^c^	.44	—	.006	<.001

^a^HIV: human immunodeficiency virus.

^b^RSV: relative search volume.

^c^Statistical significance was defined as *P*<.05.

## Discussion

### Principal Findings

Both visual inspection and joinpoint analysis showed a positive correlation between health campaigns and people’s search behaviors for the “ColonCancerCheck” and “Anti-Marijuana” campaigns. Although these associations were moderate, the results support previous studies, suggesting that infoveillance can measure the success of a campaign in driving information seeking behaviors in a population [10,32]. The “ColonCancerCheck” campaign was effective in generating online activity potentially because of the physicians being the point of contact to engage their target audience. As a result, this focused approach remained most relevant to at-risk populations, thus generating higher interest and information seeking behaviors. The impact of the “Anti-Marijuana” campaign on Google search activity may be due to the younger target audiences. The campaign aimed to educate adolescents about the negative effects of marijuana use on health. Fittingly, this target audience is also the primary user of the Web, which could have led to higher search activity for marijuana use information during the campaign period. However, no associations were found for the “Make Health Last” and “End HIV Stigma” campaigns. The null findings may be due to the differences in campaign type, frequency, and duration. Likewise, these same factors may understate the moderate associations observed for the “ColonCancerCheck” and “Anti-Marijuana” campaigns. Glynn et al [10] found positive associations between an annual breast cancer awareness campaign and related search activity in the United States. In their study, the authors observed a consistent increase in the level of Google search activity related to breast cancer during the month of October, which coincides with the annual breast cancer awareness month [10]. The success of breast cancer awareness campaign in generating interest in the population, as measured by search activity on Google, may be easier to observe because of the recurrent pattern of the campaign. The campaigns examined in our study, on the other hand, were held only once for a specific period of time. Thus, the nonrepeating nature of the campaigns compared with the annual breast awareness campaign may have contributed to the little or no effect on Google search activity.

Another difference that may have implications on the findings is the length of the campaign period. Other studies examined more impulsive interventions and their immediate influence on online activity. Noar et al [23] looked at pancreatic cancer announcements from public figures and their influence on media and search query outcomes. From their study, they found a positive association between pancreatic cancer announcements, such as a diagnosis or death, and cancer information seeking behavior in the US population. In comparison, the durations of the campaigns in our study ranged from 3 months to 1 year. Consequently, the long durations of the campaigns may have weakened the observed effect of the campaign on generating interest and information seeking behaviors.

Despite past successes of linking Web-based search activity and infectious disease outbreaks, little is known about the relationship between Web-based search activity and chronic diseases. In this study, the disease rates of colorectal cancer, HIV infection, marijuana use, and stroke showed significant correlation with Google search activity. Although disease trends have not been studied yet in literature, these findings are consistent with other related studies. In one study, the state-specific variance in stroke prevalence was shown to be related to the search query data of the specific state [14]. Google search activity was also positively associated with suicide rates in the United States [18]. Stroke, HIV, and substance use showed positive associations between disease rates and search volume. This means that more individuals with the disease would result in a corresponding increase in search activity observed on Google. Individuals may seek information on the Web before consulting a health care provider for reasons such as obtaining information about disease symptoms, diagnosis, or treatments [33]. Because of increasing Internet accessibility, more people are likely to engage in regular information seeking behaviors than in the past. Interestingly, colorectal cancer had a negative association with Web activity, which suggests that fewer individuals living with colorectal cancer would result in higher search activity on Google Trends. This inverse relationship between disease rates and online activity has not been reported in the literature. Although the reason behind this inverse relationship is not known, one possible explanation may be the decreased awareness of and interest toward colorectal cancer in Canada. In addition, cancer rates differ among the provinces and territories and an aggregate assessment may mask the true relationship between colorectal cancer prevalence and Internet search activity. Furthermore, due to the limited availability of information in scholarly journals and gray literature, colorectal cancer rates were only obtained from 2004 to 2010. It would be beneficial to assess the disease rates and search activity with more updated data to determine if the negative correlation remains persistent with time. Nonetheless, this relationship between monitoring cases of colorectal cancer and Web-based search activity should be further studied in order to confirm these findings.

### Limitations

Limitations of the study include the limited availability of information about the campaigns as well as disease statistics. Publicly available data on the prevalence of HIV, stroke, marijuana use, and colorectal cancer were limited. Ideally, disease prevalence data for the entire 2004 to 2015 period would provide the best Pearson correlation estimate. In this study, prevalence data were only available for HIV. Analyzing prevalence data would capture both individuals with newly diagnosed disease and individuals who are still currently living with the disease. Both groups should be considered because they are both potential users of the Internet who may seek more information about their disease. Compared with incidence data, this statistic only observes new cases of the disease over a period of time. Therefore, it neglects the second group of individuals who may still turn to the Web to seek health information about the disease. Consequently, individuals included in the incidence data would not be completely representative of those who are likely to use the Internet to learn about their disease.

A second limitation of this study is the primary use of Google Trends to collect and assess search activity. Although Google is currently the only search engine to offer a data analytics tool that is accessible to the public, there are biases present in using Google Trends. Because Google makes up 78% of the global market share [30], generalizability of the findings could still be maintained; however, this study still does not account for the remaining population who use other search engines such as Bing and Yahoo! [23]. Furthermore, crowdsourcing of data from the online community offers many advantages, such as readily available, easily accessible, and copious data to study. Active crowdsourcing, such as reporting flu symptoms to a Web-based tracking system, or passive crowdsourcing, such as browsing the Web, would both come with inherent biases, which must be acknowledged [34]. This pertains to the volunteer bias in which individuals who use Google as their primary search engine were found to be typically younger, have higher income, and come from larger households [35]. These characteristics may act as confounders in this study as this specific population is less likely to develop the diseases, which in turn reduces their information seeking behaviors toward the diseases.

Finally, the general limitation in studying Internet search behaviors is the uncontrollable factors that can also affect search activity. Possible confounding variables include news events such as a disease-related death of a public figure, social media influence of a public figure, and other health campaigns held during the same time period. However, this is a classic limitation of conducting an observational study in which it would be impossible to discern a cause-effect relationship.

### Future Research

Further research is necessary to study associations between health campaigns and search activity as well as associations between disease rates and search activity on the Web. First, although the effect of health campaigns on Internet search activity was not established in this study, a recommendation for future studies is to examine the effects of recurrent health campaigns. Because positive findings have been reported for breast cancer awareness initiatives in the United States by Glynn et al [10], future studies could assess similar initiatives such as Movember for prostate cancer and their impact on search activity on the Web. Second, other analytics and statistical methods could be used to determine the effects of health campaign implementation and Internet search activity outcomes. Digital marketing techniques such as Buzzmonitor are used by organizations to track their brand popularity and “buzz” in the Web-based community [36,37]. Finally, big data are generated from many different sources that can also be studied to evaluate effectiveness of health campaigns. With the gaining popularity of social media platforms, such as Facebook, Twitter, and Instagram, among public health organizations, these data may offer potential answers to better understand the effects of health campaigns on the Web-based community. In a study by Xu et al [38], they evaluated health awareness campaigns using application programming interface to collect Twitter information. Their investigation showed fluctuations in the frequency of cancer terms on Twitter during the months of cancer awareness for breast, prostate, and lung cancers, namely, September, October, and November [38]. Similarly, these applications are transferable to health campaigns seeking related outcomes such as campaign awareness and health education. Thus, additional studies exploring new techniques and big data may help support the use of infoveillance in health campaign evaluation [36,37].

Furthermore, campaigns that focus more on increasing awareness may be more appropriate to study because information-seeking behavior is the target outcome of such campaigns. For example, campaigns using advertisements to deliver their message compared with those handing out screening kits would be more relevant for engaging people on the Internet. Thus, selecting the right campaign will be an important factor to consider when studying search behaviors on Google Trends.

Application of Google Trends to provide indication of disease rates was shown to be promising in this study. However, future studies are still warranted in order to strengthen this correlation. First, disease prevalence rates should be examined instead of disease incidence rates because prevalent numbers would capture all individuals who are most likely to seek information regarding the disease on the Internet. In particular, diseases that have shown upward and downward trends in prevalence rates over a period of time will be best to study. With fluctuations in both positive and negative directions, it would test whether the search activity would also follow the fluctuations in both positive and negative directions. If both the disease rates and the Internet search activity agree within the same time frame, this correlation would strengthen the use of infodemiology in monitoring diseases in a population.

### Conclusions

In this study, analysis of Internet search data showed significant relationships between health campaigns and information seeking behaviors in Canada for colorectal cancer and substance use but not for HIV and AIDS and stroke. The outcomes of the “ColonCancerCheck” and “Anti-Marijuana” campaigns were consistent with previous studies. Possible reasons for the discrepant findings from the “Make Health Last” and “End HIV Stigma” campaigns include differences in campaign type, frequency, and duration. However, the use of Web-based search data on digital disease monitoring remains promising. The study found significant associations between search activity and disease prevalence or incidence rates. Further studies are needed to validate the reliability of using Google Trends for health research purposes.

## Appendix

Multimedia Appendix 1 List of search terms reviewed for inclusion in the study.

Multimedia Appendix 2 Results from joinpoint analysis for colorectal cancer.

Multimedia Appendix 3 Results from joinpoint analysis for stroke.

Multimedia Appendix 4 Results from joinpoint analysis for substance abuse.

Multimedia Appendix 5 Results from joinpoint analysis for human immunodeficiency virus and AIDS.

## Abbreviations

AIDS: acquired immunodeficiency syndrome
HIV: human immunodeficiency virus
RSV: relative search volume
