# Supplementary material for: Disease Monitoring and Health Campaign Evaluation Using Google Search Activities for HIV and AIDS, Stroke, Colorectal Cancer, and Marijuana Use in Canada: A Retrospective Observational Study
Source: JMIR Public Health Surveill. 2016 Oct 12;2(2):e156. doi: 10.2196/publichealth.6504 (PMC5081479; doi:10.2196/publichealth.6504)
Supplement: Multimedia Appendix 1 [file publichealth_v2i2e156_app1.pdf]

Multimedia Appendix 1: List of search terms reviewed for inclusion in the study

Search category	Colorectal cancer	HIV and AIDS	Stroke	Substance abuse
**Health related search terms**	Colorectal cancer + colorectal diagnosis + colorectal screening + colorectal cancer screening + colon cancer + colon cancer symptoms	HIV + AIDS + Human immunodeficiency virus + acquired immunodeficiency virus + HIV symptoms + HIV diagnosis + AIDS symptoms + AIDS diagnosis + HIV contraction	Stroke + Stroke symptoms + FAST + stroke onset	Marijuana use + drug use + marijuana side effects + marijuana effects
**Google Trends suggested search terms**	Colon cancer; Symptoms colorectal cancer; Colorectal cancer screening; Colorectal cancer Canada; Association cancer colorectal; Colorectal cancer treatment; Symptoms cancer colorectal; Rectal cancer; Bowel cancer; Colon cancer symptoms	Hiv aids; Hiv/aids Canada; Hiv/aids in Canada; Hiv/aids in Africa; What is hiv/aids; Hiv/aids symptoms; Hiv/aids treatment; Hiv and aids; Hiv/aids statistics; Hiv/aids prevention	Heart stroke; Heart; Heart and stroke; Heart stroke lottery; Heart stroke calendar; Heart stroke foundation; The stroke; 2 stroke; stroke symptoms; 4 stroke	Canada cannabis; Marijuana; Cannabis culture; Medical cannabis; Cannabis oil; Cannabis seeds; Cannabis in Canada; Cannabis plant, Canabis; Le cannabis
**Campaign search terms**	Colon cancer check + CCC	BC persons with AIDS society + end HIV stigma	Heart and Stroke foundation + FAST commercial + make health last	Drugs Not 4 Me + drugsnot4me + drugs not for me
