# Supplementary material for: Disease Monitoring and Health Campaign Evaluation Using Google Search Activities for HIV and AIDS, Stroke, Colorectal Cancer, and Marijuana Use in Canada: A Retrospective Observational Study
Source: JMIR Public Health Surveill. 2016 Oct 12;2(2):e156. doi: 10.2196/publichealth.6504 (PMC5081479; doi:10.2196/publichealth.6504)
Supplement: Multimedia Appendix 2 [file publichealth_v2i2e156_app2.pdf]

Multimedia Appendix 2: Results from joinpoint analysis for colorectal cancer

Statistical outputs		5 week period	10 week period	15 week period
**Segment 1 (week)**		1-3	1-12	1-14
	Slope, RSV[a]/week (95% CI)	9.86 (7.7 to 12.0)	2.22 (2.1 to 2.3) −0.27 (−0.4 to −0.2)	
	P value[b]	.14	<.001	.50
**Segment 2 (week)**		3-12	12-23	14-22
	Slope, RSV/week (95% CI)	0.21 (0.03 to 0.4)	−1.62 (−1.7 to −1.5)	0.04 (−0.1 to 0.2)
	P value[b]	.54	<.001	.95
**Segment 3 (week)**		12-15	23-46	22-25
	Slope, RSV/week (95% CI) -5.00 (−7.11 to −2.6)	0.07 (0.04 to 0.10)	−4.59 (−6.2 to −3.0)	
	P value[b]	.45	.58	.45
**Segment 4 (week)**		15-36	—	25-56
	Slope, RSV/week (95% CI)	0.01 (−0.03 to 0.05)	—	−0.07 (−0.09 to −0.05)
	P value[b]	.97	—	.35

[a]RSV: relative search volume.

[b]Statistical significance was defined as $P < .05$.
