# Supplementary material for: Disease Monitoring and Health Campaign Evaluation Using Google Search Activities for HIV and AIDS, Stroke, Colorectal Cancer, and Marijuana Use in Canada: A Retrospective Observational Study
Source: JMIR Public Health Surveill. 2016 Oct 12;2(2):e156. doi: 10.2196/publichealth.6504 (PMC5081479; doi:10.2196/publichealth.6504)
Supplement: Multimedia Appendix 3 [file publichealth_v2i2e156_app3.pdf]

Multimedia Appendix 3: Results from joinpoint analysis for stroke

Statistical outputs		5 week period	10 week period	15 week period
**Segment 1 (week)**		1-3	1-4	1-9
	Slope, RSV[a]/week (95% CI)	−12.87 (−14.3 to −11.5)	−2.29 (−3.1 to −1.5) −0.32 (−0.5 to −0.2)	
	P value[b]	.002	.38	.51
**Segment 2 (week)**		3-13	4-7	9-12
	Slope, RSV/week (95% CI)	−1.33 (−1.4 to −1.2)	12.62 (10.8 to 14.5)	11.63 (10.2 to 13.0)
	P value[b]	0.001	.04	.03
**Segment 3 (week)**		13-27	7-10	12-15
	Slope, RSV/week (95% CI) 0.15 (0.1 to 0.2)	−8.54 (−10.4 to −6.7)	−7.92 (−9.9 to −6.5)	
	P value[b]	.22	.15	0.13
**Segment 4 (week)**		—	10-37	15-47
	Slope, RSV/week (95% CI)	—	−0.28 (−0.3 to −0.3)	−0.38 (−0.49 to −0.37)
	P value[b]	—	<.001	<0.001

[a]RSV: relative search volume.

[b]Statistical significance was defined as $P < .05$.
