# Supplementary material for: Disease Monitoring and Health Campaign Evaluation Using Google Search Activities for HIV and AIDS, Stroke, Colorectal Cancer, and Marijuana Use in Canada: A Retrospective Observational Study
Source: JMIR Public Health Surveill. 2016 Oct 12;2(2):e156. doi: 10.2196/publichealth.6504 (PMC5081479; doi:10.2196/publichealth.6504)
Supplement: Multimedia Appendix 4 [file publichealth_v2i2e156_app4.pdf]

Multimedia Appendix 4: Results from joinpoint analysis for marijuana use

Statistical outputs		5 week period	10 week period	15 week period
**Segment 1 (week)**		1-14	1–18	1-11
	Slope, RSV[a]/week (95% CI)	1.13 (1.1 to 1.2)	0.83 (0.8 to 0.9) −0.15 (−0.3 to −0.1)	
	P value[b]	<.001	<.001	0.60
**Segment 2 (week)**		14-18	18–22	11–23
	Slope, RSV/week (95% CI)	−3.81 (−4.1 to −3.5)	−3.50 (−3.9 to −3.1)	1.09 (1.0 to 1.2)
	P value[b]	<.001	.003	<.001
**Segment 3 (week)**		18-21	22–25	23–26
	Slope, RSV/week (95% CI) 4.08 (3.4 to 4.7)	3.61 (3.0 to 4.2)	−3.31 (−4.5 to −2.1)	
	P value[b]	.03	.06	0.34
**Segment 4 (week)**		21-24	25-33	26–43
	Slope, RSV/week (95% CI)	−1.57 (−1.9 to −1.3	-0.13 (−0.2 to −0.02)	0.46 (0.4 to 0.5)
	P value[b]	.06	0.67	<.001

[a]RSV: relative search volume.

[b]Statistical significance was defined as $P < .05$.
