# Supplementary material for: Disease Monitoring and Health Campaign Evaluation Using Google Search Activities for HIV and AIDS, Stroke, Colorectal Cancer, and Marijuana Use in Canada: A Retrospective Observational Study
Source: JMIR Public Health Surveill. 2016 Oct 12;2(2):e156. doi: 10.2196/publichealth.6504 (PMC5081479; doi:10.2196/publichealth.6504)
Supplement: Multimedia Appendix 5 [file publichealth_v2i2e156_app5.pdf]

**Multimedia Appendix 5:** Results from joinpoint analysis for HIV[a] and AIDS[b]

| Statistical outputs | | 5 week period | 10 week period | 15 week period |
|---|---|---|---|---|
| **Segment 1 (week)** | | 1-67 | 1-77 | 1-87 |
| | Slope, RSV[c]/week (95% CI) | −0.07 (−0.01to −0.002) | -0.06 (−0.1 to −0.05) -0.05 (−0.045 to −0.049) | |
| | *P* value[d] | .001 | <.001 | <.001 |

[a]HIV: human immunodeficiency virus.

[b]AIDS: acquired immunodeficiency syndrome.

[c]RSV: relative search volume.

[d]Statistical significance was defined as $P < .05$.
